# Characterization of volatile compounds from healthy and citrus black spot-infected Valencia orange juice and essential oil by using gas chromatography–mass spectrometry

**DOI:** 10.1016/j.fochx.2024.101374

**Published:** 2024-04-10

**Authors:** Leng Han, Guijie Li, Xuting Wang, Bo Yu, Tenghui Zhang, Yujiao Cheng

**Affiliations:** a Citrus Research Institute, Southwest University, Chongqing 400712, China; b National Citrus Engineering Research Center, Chinese Academy of Agricultural Sciences, Chongqing 400712, China; c Sichuan Dan Orange Modern Fruit Industry Co., Ltd., Sichuan 620200, China; d Chongqing Centre Testing International Group Co., Ltd., Chongqing 400712, China

**Keywords:** Citrus juice, Essential oil, Citrus black spot, Volatiles, Variable importance for the projection, Linalool (PubChem CID:6549), β-myrcene (PubChem CID:31253), d-limonene (PubChem CID:440917), 1-octen-3-ol (PubChem CID:18827), 1-octanol (PubChem CID:957), terpinen-4-ol (PubChem CID:11230), α-terpineol (PubChem CID:17100), citronellol (PubChem CID:8842), ethyl butyrate (PubChem CID:7762), and γ-terpinene (PubChem CID:7461).

## Abstract

Citrus black spot (*Phyllosticta citricarpa,* CBS) is an important fungal disease that causes rind blemishes and affects quality of citrus fruits. The response of citrus to CBS in terms of volatiles was evaluated using molecular sensory science approaches. Fifty and twenty-one volatiles were identified in the orange juice and essential oil samples, respectively, via gas chromatography–mass spectrometry (GC–MS). The total volatile content in the samples increased after CBS infection, especially in the severe-infection group (SEG) juice and moderate-infection group (MOG) essential oil, which reached the highest levels. CBS enhanced floral, fruity, and off-flavor aromas and decreased the green aroma in citrus juice. Citrusy, floral, and green aromas increased in the CBS-infected essential oil. Six/five potential markers were screened in citrus juice/essential oil, respectively using the orthogonal partial least-square discriminant analysis (OPLS-DA) model. The changes in aroma profile and the difference in infection levels in citrus were attributed to these odorants.

## Highlights

• 50/21 volatiles including 35/12 odorants identified in orange juice/essential oil.
• Citrus black spot (CBS) disease increases total volatile content in orange.
• Floral & fruity aromas and off-flavor increase; green aroma declines in CBS juice.
• Ten potential markers were screened based on multivariate analysis.

## Graphical abstract

**Figure:**
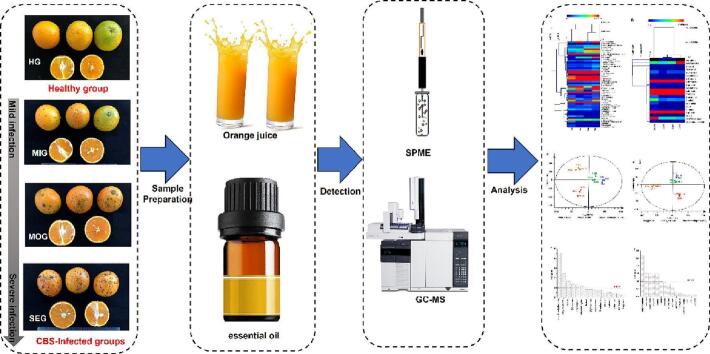

## 1 Introduction

Citrus (*Citrus reticulata* Blanco), mainly including orange, mandarin, pomelo and so forth, is one of the important fruit varieties in the world and is cultivated in >140 countries. According to citrus report from the United States Department of Agriculture (2024), the global production of citrus can reach about 104 million tons (Citrus: World markets and trade, 2024). Citrus fruits are popular among consumers worldwide due to their various nutrients and unique flavor. Except for consuming fresh fruits, citrus is often processed into products such as juice, canned fruit and essential oils. However, during growth, citrus can be easily affected by fungi, bacteria, or viruses, resulting in a variety of diseases, such as citrus black spot (CBS), huanglongbing (HLB), and citrus decline. CBS is an important disease caused by the fungus *Guignardia citricarpa* (Kiely, 1948) and was first reported by Benson (1895). The parasitosis of the asexual conidium of citrus black spot is weak, and the sexual ascospore is the main source of infection. CBS is a foliar and fruit disease. The pathogen does not show symptoms in the young fruit stage until the fruit begins to show disease symptoms in the epidermis from the expansion stage to the maturity stage. This disease is found in the most citrus growing areas around the world and can cause fruit different spotty symptoms including hard spot, freckle spot, cracked spot, false melanosis spot and virulent spot. The most typical symptom is a hard spot, which appears when the citrus fruit is ripe and is generally round and depressed, with a diameter of 3–10 mm. The center of these lesions is tan to gray with a distinct or prominent brick-red to dark brown margin (Silva Junior, Feichtenberger, Spósito, & Bassanezi, 2016). All citrus species except for sour orange, lime, and pummelo are vulnerable to CBS, and infected fruit peels develop black spots (Miles et al., 2019). The presence of CBS lesions causes high economic losses in the fresh fruit market due to rind blemish. In the European Union, CBS is listed as an A1 quarantine pest, seriously affecting global sales of fresh citrus fruits (EPPO, 2020). CBS disease increased the total soluble solid (TSS) and total acid (TA) values of sweet orange fruits, but the internal quality still satisfies international quality standards (Brentu et al., 2012; UNECE, 2010). Moreover, the CBS disease does not affect the flesh of citrus fruits, the infected CBS citrus fruits can still be suitable for processing (Futch, 2011; Malik et al., 2021).

Volatile compounds (VOCs), which are important secondary metabolites in citrus fruits, are mainly present in the peel and pulp and have an important influence on the overall flavor of citrus. Citrus essential oil is obtained from the peel portion and is mainly composed of mono- and sesquiterpene hydrocarbons and their oxygenated derivatives (Singh, Singh, Kaur, & Yadav, 2021). Ethyl butyrate, myrcene, limonene, 4–isopropyltoluene, linalool, and decanal were proven to be the key aroma compounds in the five sweet orange essential oils based on their high odor active values (OAVs) (Shui et al., 2019). In addition to aroma properties, citrus essential oil exhibits antioxidant, antidiabetic, and insecticidal, properties (Bora, Kamle, Mahato, Tiwari, & Kumar, 2020). Moreover, ethyl 2-methylpropanoate, ethyl butanoate, (*S*)-ethyl 2-methylbutanoate, 3a,4,5,7a-tetrahydro-3,6-dimethyl-2(3H)-benzofuranone, (*Z*)-hex-3-enal and decanal, which present fruity, grassy and citrus-like attributes, have been identified as the most potent odorants in Valencia late and Navel orange juice (Buettner & Schieberle, 2001). The flavor quality of citrus juice and essential oils has a significant impact on consumer acceptance of products.

Previous studies have reported that diseases significantly altered the volatile profile of citrus juice and essential oils (da Cruz, Plotto, Ferrarezi, Leite Junior, & Bai, 2023; Dala-Paula et al., 2018). Symptomatic Valencia orange juice was reported to have a higher concentration of γ-terpinene and linalool and a lower concentration of a-terpinolene and ethyl butanoate than a control sample (Dagulo et al., 2010). The aldehyde content in symptomatic orange juice was found to be higher than that in the control samples (Dagulo et al., 2010). Moreover, the difference in aroma between symptomatic and asymptomatic orange oil samples was also proven by panel tests (Xu, Sims, Etxeberria, & Schneider, 2017). However, studies on the effects of disease on the flavor of citrus juice or essential oil have only focused on HLB, and there have been few studies on the effects of CBS and the volatile changes between healthy and CBS-infected orange juice or essential oil. The related flavor study depended on the levels of CBS severity could provide a theoretical basis for the selection of raw materials and citrus juice/essential oil processing. Therefore, the main purposes of this study were to identify VOCs, monitor changes in different CBS-infected levels of Valencia orange juice or peel oil by using gas chromatography–mass spectrometry (GC–MS), determine the influence of VOCs on the overall flavor profile by applying OAVs, and investigate the correlation of aroma compounds with healthy or different levels of CBS severity.

## 2 Material and methods

### 2.1 Sample preparation

**Fig. 1:**
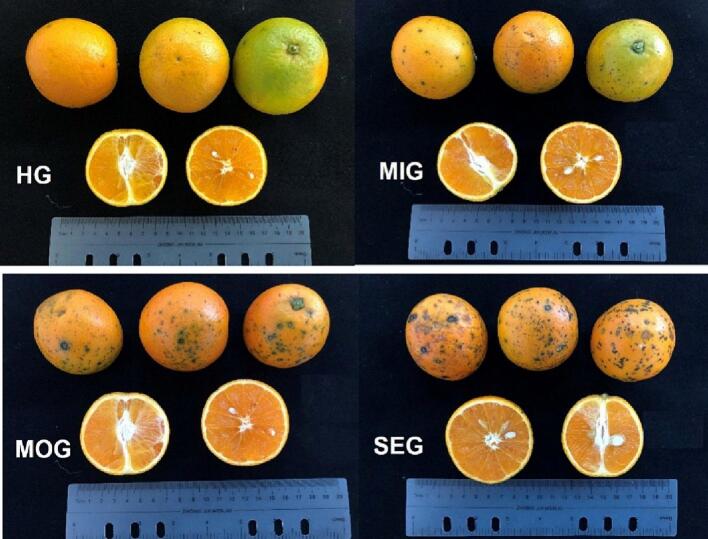
Fruit appearance of HG, MIG, MOG, and SEG citrus fruits.

“Valencia” oranges (*Citrus sinensis* (L.) Osbeck) were obtained from orchards on June 08, 2022 (Xiema, Beibei district, Chongqing, China). Citrus fruits were harvested from visually CBS symptomatic trees (displaying black spots on the fruit peel) and visually healthy trees (no CBS symptoms). Thirty fruits were picked from one tree and the experiment was repeated with three trees. Different CBS-infected fruits were picked from symptomatic trees and classified into a mild-infection group (MIG), a moderate-infection group (MOG), and a severe-infection group (SEG) according to the size and number of black spots on the fruit peel by using Bold extract software (Fig.1). Citrus fruits obtained from the visually healthy trees were designated the healthy group (HG). By integrating data from a large number of CBS citrus fruits, when the area proportion of black spots to the whole citrus fruits is 0,0–4%, 4–7%, and >7%, it is defined as HG, MIG, MOG, and SEG (Fig.S1). The essential oil extraction and juice preparation of the orange fruits in symptomatic and CBS noninfected groups were performed using the same processing method. The peel essential oil was prepared by a cold-pressing method as described previously with modifications (Njoroge, Ukeda, & Sawamura, 1996). The citrus fruits were washed with water and dried, and the flavedo layer was peeled with a rotary peeler. The oil emulsion was pressed by a CJ3000 blender (Braun Co., Kronberg, Germany) and centrifuged at 13400 ×*g* for 10 min at 4 °C. Then, the collected samples were frozen for two days at −20 °C, the lower layer of precipitation was discarded, and the upper layer of clear liquid was the cold-pressed oil for the study. The peeled citrus fruits were cut in half and hand-squeezed into juice by a blender (CJ3000, Braun Co., Germany). The juice was filtered through an 80-mesh filter and kept at −20 °C until further analysis.

### 2.2 Reagents and chemicals

Cyclohexanone (99%) was purchased from Aladdin Industrial Co. (Shanghai, China). n-Alkanes (C5–C20) were purchased from Sigma-Aldrich Co. (Shanghai, China).

### 2.3 Verification of CBS lesion diagnosis

CBS lesions was verified according to previously reported studies (Moyo et al., 2020; Schreuder et al., 2018). A quantitative polymerase chain reaction (qPCR) protocol was used to test the 8 lesions and verify the accuracy of the visual diagnosis of CBS lesions caused by P. citricarpa. DNA was extracted from the representative CBS lesion described by the hexadecyl trimethyl ammonium bromide (CTAB) method. A rapid detection system was established using the screened specific primers GCP and MightyAmp @ DNA polymerase. The DNA extracted from the citrus sample was used as the template, and the pure cultured genomic DNA of the citrus black spot pathogen (NHB1) was used as the control. The PCR product was detected by 1.0% agarose gel electrophoresis.

### 2.4 Solid phase microextraction (SPME)

The VOCs from citrus juice or essential oil were separated and enriched by the SPME method described in a previous report with modification (Cheng, Li, Wu, Liang, & Wang, 2022; Li et al., 2020). Citrus juice (5 mL), sodium chloride (1.8 g), 2.5 μL internal standard (cyclohexanone 9.423 mg/mL), and a stir bar were placed into a 20 mL glass vial that been flushed with nitrogen gas for 10 s. The vials were capped with Teflon-faced silicone septa. The vial with citrus juice was equilibrated at 40 °C for 20 min. A 2 cm SPME fiber coated with DVB/CAR/PDMS (Supelco, Bellefonte, PA) was used to extract VOCs for 30 min with instant agitation. Essential oil (50 μL) and 2.5 μL internal standard (18.85 μg/mL cyclohexanone) were placed into a 20 mL glass vial that had been flushed with nitrogen gas for 10 s. The vial with essential oil was equilibrated at 40 °C for 30 min. A 1 cm SPME fiber coated with CAR/PDMS (Supelco, Bellefonte, PA) was used to extract VOCs for 15 s. The desorption of VOCs enriched in the fiber was conducted in the injector of GC at 230 °C for 5 min.

### 2.5 Identification and quantification of volatiles

GC analysis was performed for citrus juice or essential oil using an Agilent 7890B GC equipped with a 5977 A MS detector. A DB-5 column (30 m × 0.25 mm id × 0.25 μm) was used to separate VOCs. Helium was used as the carrier gas and applied at a rate of 1.2 mL/min. The initial oven temperature was held at 35 °C for 3 min, then raised to 203 °C at 6 °C /min, and finally increased to 243 °C at 10 °C /min and held for 3 min. The transfer line and ion source temperature were set to 280 °C and 230 °C, respectively. The mass spectra were obtained in electron impact (EI) mode at 70 eV, and full scanning was performed over a range of *m*/*z* 33–500 amu. VOCs were identified by matching their mass spectra with the total ion chromatography (TIC) from the mass spectral library (NIST11, W10N14), and by comparing the linear retention index (LRI) from the related references. LRI values were calculated by using alkane standards (C5-C20). Three replicate samples were used for the flavor analysis. The internal standard of cyclohexanone was used to semi-quantify the VOCs in the citrus juice or essential oil. The unit of volatiles should be equivalents of mg of the internal standard. OAVs were calculated by the ratio of concentration to threshold. The olfactory threshold values were obtained from literature studies (Table S1).

### 2.6 Statistical analysis

Graphics were created using Origin 7.5 (OriginLab, Northampton, MA). The heatmap was constructed by means of multiple experiment Viewer. The multivariate analysis, involving principal component analysis (PCA), and orthogonal partial least-square discriminant analysis (OPLS-DA), was performed in SIMCA 14.1 software (Umetrics, Umeå, Sweden).

## 3 Results and discussion

### 3.1 Identification and quantification of VOCs in both healthy and CBS-infected orange juice

SPME has the characteristics of being solvent-free and allowing rapid extraction and is extensively applied in food analysis. The extraction efficiency of polar and nonpolar compounds in the samples varied depending on the type of fiber coating. A three fiber DVB/CAR/PDMS composite was preferentially applied for the extraction of volatiles in fruit juice such as citrus juice (Rega, Fournier, & Guichard, 2003), watermelon juice (Liu, He, & Song, 2018) and mango juice (Wibowo, Grauwet, Gedefa, Hendrickx, & Van Loey, 2015) etc. As shown in Table 1, a total of 50 volatiles were positively identified by GC–MS in the orange juice based on a combination of both mass spectra and LRI value matching: 18 alcohols, 15 terpenes, 10 esters, 5 ketones and 2 aldehydes. Numerically, alcohols comprised 36% of all volatiles in the orange juice, followed by terpenes (30%), esters (20%), ketones (10%), and aldehydes (4%). Ethyl isobutyrate, which has a fruity odor, was not detected in CBS noninfected orange juice, which was the major aromatic fraction type difference between symptomatic and CBS noninfected orange juice. This compound has been previously reported in an orange juice study (Plotto, Margaria, Goodner, & Baldwin, 2008).

**Table 1:** Identification of VOCs in both healthy and different CBS infection levels in citrus juice and essential oil.

Compound	RI (reference)^a^	RI (calculated)	identification	Detected site
**Terpenes**				
α-thujene	928	929	RI, MS	peel
α-pinene	934	938	RI, MS	juice, peel
camphene	953	950	RI, MS	peel
sabinene	973	977	RI, MS	peel
β-pinene	978	980	RI, MS	peel
β-myrcene	990	996	RI, MS	juice, peel
cosmene	1006	1011	RI, MS	juice
δ-3-carene	1011	1015	RI, MS	peel
p-1,3,8-menthatriene	1112	1039	RI, MS	juice
d-limonene	1031	1043	RI, MS	juice, peel
β-ocimene	1043	1052	RI, MS	peel
γ-terpinene	1059	1067	RI, MS	juice, peel
terpinolene	1090	1092	RI, MS	peel
4(5)-carene	–	1097	MS	juice
copaene	1376	1377	RI, MS	juice
β-elemene	1391	1392	RI, MS	juice
β-caryophyllene	1420	1422	RI, MS	juice
β-selinene	1487	1450	RI, MS	juice
β-panasinsene	–	1476	MS	juice
valencene	1494	1496	RI, MS	juice
eremophilene	1490	1506	RI, MS	juice
(−)-α-panasinsen	1527	1523	MS	juice
**Alcohols**				
ethanol	537	575	RI, MS	juice
2-methyl-3-buten-2-ol	612	615	RI, MS	juice
1-penten-3-ol	682	673	RI, MS	juice
3-methylbutanol	737	723	RI, MS	juice
2-methylbutanol	740	727	RI, MS	juice
pentanol	765	757	RI, MS	juice
(Z)-3-hexenol	856	854	RI, MS	juice
(*E*)-2-hexenol	860	866	RI, MS	juice
hexanol	869	872	RI, MS	juice
1-heptanol	969	976	RI, MS	juice
1-octen-3-ol	980	984	RI, MS	juice
1-octanol	1071	1077	RI, MS	juice, peel
linalool	1100	1107	RI, MS	juice, peel
p-mentha-2,8-dien-1-ol	1121	1128	RI, MS	juice
terpinen-4-ol	1178	1189	RI, MS	juice
α-terpineol	1191	1202	RI, MS	juice
cis-carveol	1229	1225	RI, MS	juice
citronellol	1228	1231	RI, MS	juice
**Aldehydes**				
hexanal	801	791	RI, MS	juice
nonanal	1103	1111	RI, MS	juice, peel
citronellal	1155	1154	RI, MS	peel
decanal	1205	1203	RI, MS	peel
neral	1239	1238	RI, MS	peel
citral	1284	1265	RI, MS	peel
**Esters**				
ethyl acetate	613	623	RI, MS	juice
methyl butanoate	724	709	RI, MS	juice
ethyl isobutyrate	754	747	RI, MS	juice
ethyl butyrate	801	793	RI, MS	juice
ethyl 2-methylbutyrate	847	845	RI, MS	juice
methyl hexanoate	929	927	RI, MS	juice
ethyl hexanoate	999	1006	RI, MS	juice, peel
hexyl acetate	1012	1019	RI, MS	juice
ethyl 3-hydroxyhexanoate	1129	1133	RI, MS	juice
ethyl octanoate	1197	1199	RI, MS	juice
octyl acetate	1209	1207	RI, MS	peel
**Ketones**				
2-pentanone	677	675	RI, MS	juice
6-methyl-5-hepten-2-one	985	991	RI, MS	juice
(+)-carvone	1256	1248	RI, MS	juice
α-ionone	1428	1425	RI, MS	juice
geranylacetone	1451	1444	RI, MS	juice
**others**				
(E)-limonene oxide	1140	1141	RI, MS	peel

a RI(reference)come from the library: https://www.vcf online.nl/VcfCompoundSearch.cfm, http://www.odour.org.uk/index.ht mL and https://webbook.nist.gov/chemistry/name-ser/

**Fig. 2:**
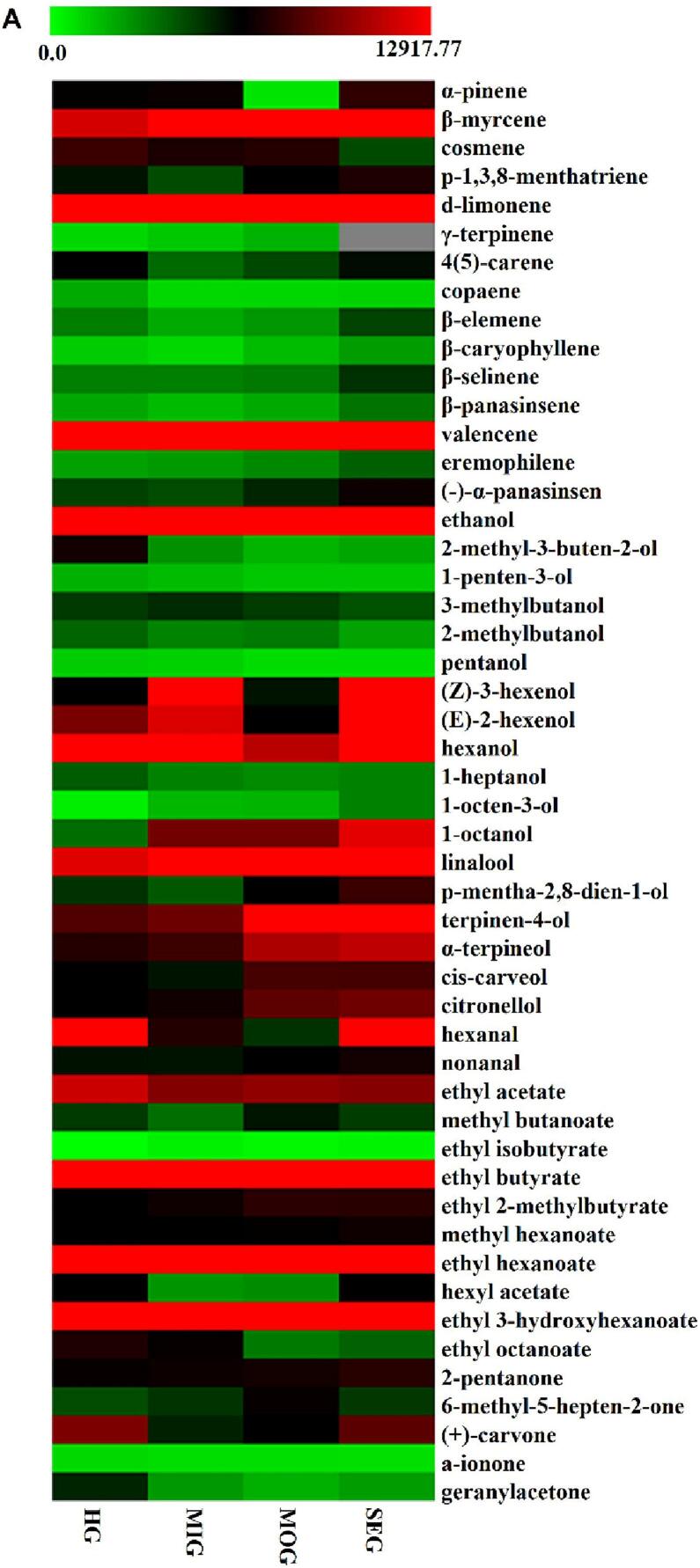

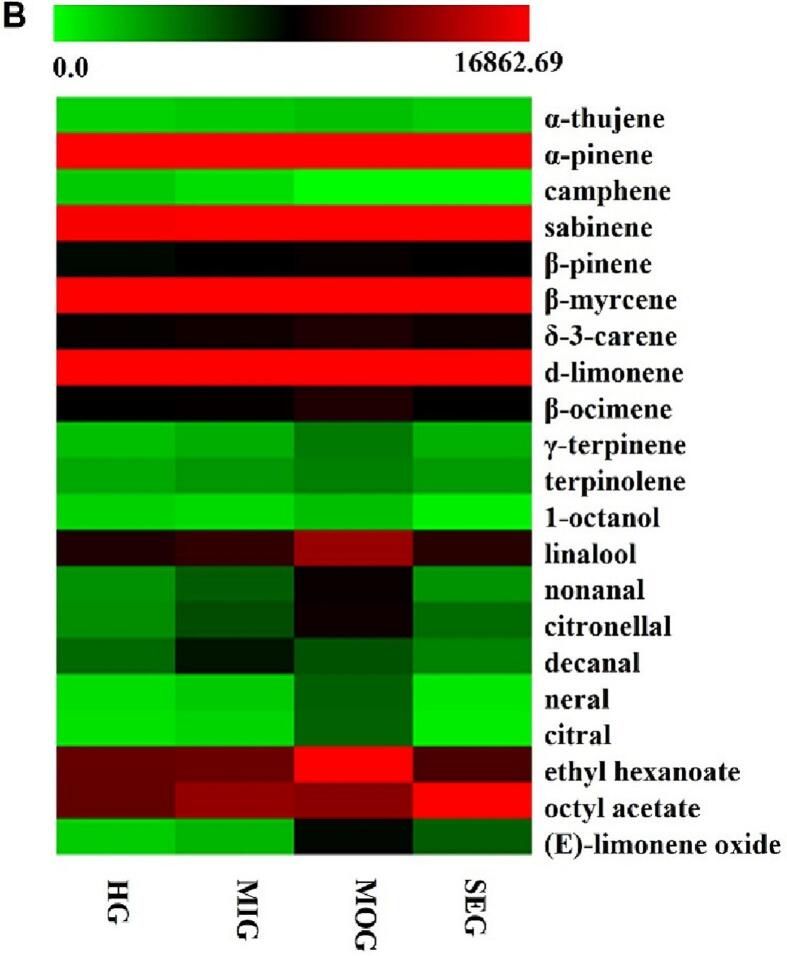
Heatmap illustrating the difference in VOCs between healthy and black spot orange juice and essential oil.

The relative concentrations of volatiles were calculated by means of the internal standard method. As shown in Fig.S2A, CBS affected the content of volatiles in orange juice. The SEG orange juice (992.61 ± 82.33 mg/L) had the highest content of volatiles, followed by the MIG (727.87 ± 56.53 mg/L), MOG (711.95 ± 56.53 mg/L) and HG (674.33 ± 82.17 mg/L) samples. The content of total volatiles in the symptomatic groups was higher than that in the CBS noninfected group. The results showed that CBS induced the production of volatiles of secondary metabolites in orange pulp. The difference in the contents of volatiles also proved the defense response of the host citrus to CBS. When orange was infected with CBS, the contents of terpenes and alcohols in orange juice increased, while the contents of esters and aldehydes decreased. Fifteen volatiles, namely, 6 alcohols (ethanol, 2-methyl-3-buten-2-ol, 1-penten-3-ol, 2-methylbutanol, pentanol, 1-heptanol), 3 terpenes (cosmene, 4(5)-carene, copaene), 3 ketones ((+)-carvone, a-ionone, geranylacetone), 2 esters (ethyl acetate, ethyl octanoate), and 1 aldehyde (hexanal), had lower concentration in the symptomatic group than in the CBS noninfected group. However, the results also showed that the concentrations of thirteen volatiles, namely, 5 terpenes (d-limonene, valencene, β-myrcene, eremophilene and γ-terpinene), 6 alcohols (linalool, terpinene-4-ol, a-terpineol, citronellol, 1-octanol and 1-octen-3-ol), 1 ester (ethyl butyrate), 1 ketone (2-pentanone), continuously increased from HG to MIG to MOG to SEG (Fig.2A). In this study, the increase in the terpenes content indicated the resistance and self-protection of the host citrus to the fungus *Guignardia citricarpa*.

The terpenes class, accounting for 57.76% ∼ 60.98% of the total content, was the most abundant chemical class in orange juice. The major terpenes in orange juice were d-limonene, followed by valencene and β-myrcene. The concentrations of three volatiles increased in the juice of CBS-infected fruits. Previous studies have also observed that HLB disease stimulates an increase in the content of d-limonene and β-myrcene in grapefruit fruits (da Cruz et al., 2023). It has been reported that some terpenes, especially d-limonene, have a good effect against fungi or bacteria, such as the pathogens *Candida tropicalis, Bacillus subtilis, Aspergillus niger,* and *Listeria monocytogenes* (Aggarwal et al., 2002; Han, Sun, & Chen, 2019; Yu et al., 2022). Alcohols were the secondary metabolites detected in this study. Higher alcohols content, such as ethanol, hexanol, and linalool, were detected in the CBS noninfected group and SEG group. From the healthy group to groups with different levels of CBS severity, only the concentration of linalool compound in orange juice showed an increasing trend. It may be related to fungus *Guignardia citricarpa* enzymes or metabolites, or a stress response caused by fungal damage, that increased the activity of terpenes synthases (TPs) in citrus fruits. TPs were the vital terminal enzymes that catalyze the formation of monoterpene linalool. The overexpression of PpTPS3 could significantly increase in the content of free and glycosidic bound linalool (Wei et al., 2021). Many studies have proven the antifungal activity of linalool, such as *Aspergillus* flavus and *P. aeruginosa*, and applied it to wheat grains for postharvest preservation (Li et al., 2022).

Moreover, the changes in two volatile compounds, a-terpineol, and terpinene-4-ol, were related to the oxidation of limonene (Perez-Lopez, Saura, Lorente, & Carbonell-Barrachina, 2006). Ethyl butyrate was the only ester whose concentration increased due to CBS infections,with an increase of 20.32% in the SEG group compared with the healthy group. The contents of aldehydes and ketones were not high, but these two classes of volatiles have important contributions to the overall flavor of orange juice. However, the concentrations of most aldehydes and ketones showed a decreasing trend in the symptomatic groups, which was consistent with a previous study (da Cruz et al., 2023).

### 3.2 Identification and quantification of VOCs in both healthy and CBS orange essential oil

As shown in Table 1, a total of 21 volatiles were extracted by SPME and identified by GC–MS from all orange essential oils, namely, 11 terpenes, five aldehydes, two alcohols, two esters, and one other class. Camphene was not detected in the MOG and SEG orange essential oils. Furthermore, the concentrations of 21 volatiles were determined using the internal reference cyclohexanone. By comparison, the contents of volatiles in the HG, MIG, MOG, and SEG essential oils were 16,423.24 mg/L, 18159.63 mg/L, 22093.41 mg/L, and 17,324.83 mg/L, respectively. The contents tended to increase first and then decrease as the level of CBS severity increased, and the concentration of volatiles was the highest in the MOG samples. As shown in Fig.S2B, terpenes (16,126.87–21,435.30 mg/L) were the predominant volatiles in the essential oil, followed by esters (215.49–391.09 mg/L), alcohols (48.94–161.21 mg/L), aldehydes (28.60–89.51 mg/L) and other classes (3.33–16.31 mg/L). Moreover, the contents of terpenes, alcohols, esters, and other classes in the symptomatic groups were higher than those in the CBS noninfected group. In the essential oil samples, mild infection with CBS increased the total content of aldehydes, while severe infection with CBS reduced it.

In this study, terpenes (16,859.60–21,435.30 mg/L) were the predominant volatiles in the essential oil, followed by esters (267.90–391.09 mg/L), alcohols (54.32–161.21 mg/L), aldehydes (27.66–89.51 mg/L) and other classes (4.79–16.31 mg/L). As shown in Fig.2B, limonene accounted for 83.82% ∼ 88.96% of the total volatile concentration and was the major volatile compound in the Valencia orange oil, followed by myrcene (4.77% ∼ 7.12%) and a-pinene (2.21% ∼ 3.13%). The percentage composition of limonene (95.6%) in Spanish Valencia orange essential oil was higher than that in a previous study (Chisholm, Jell, & Cass, 2003; Herman, Tambor, & Herman, 2016), and the quality difference may be related to variations in climate and management practices in different regions. Linalool was the major alcohol in the essential oil. Decanal was the major aldehyde in the HG and MIG essential oils, and citronellal was the main aldehyde in the MOG and SEG essential oils. Furthermore, the concentrations of thirteen volatiles (a-thujene, sabinene, β-pinene, myrcene, δ-3-carene, d-limonene, β-ocimene, γ-terpinene, terpinolene, linalool, citronellal, octyl acetate, and (*E*)-limonene oxide) in the different symptomatic level groups were higher than those in the CBS noninfected group, indicating a CBS-induced stress response. Previous reports showed a lower concentration of limonene (da Cruz et al., 2023) and linalool (Xu, Baker, Sarnoski, & Goodrich-Schneider, 2017) in HLB-infected orange peel oil. Moreover, the concentrations of 8 VOCs, namely, a-pinene, camphene, 1-octanol, nonanal, decanal, neral, citral, and ethyl hexanoate, in the SEG essential oil sample were lower than those in the HG group. There were only two volatiles (camphene and 1-octanol) in MIG and one volatile (camphene) whose contents were lower in the MOG samples than in the HG sample.

### 3.3 Citrus black spot effects on the OAVs of aroma compounds in orange juice and essential oils

To determine the contribution of a single odorant to the overall aroma, OAVs were calculated as the ratio of the concentration to odor threshold in water from references. OAVs of aroma compounds were equal to or >1 indicated that these compounds were responsible for the citrus aroma. As shown in Table 2, the total potential aroma contributor (OAVs ≥1) numbers were 33, 34, 35, and 35 in the HG, MIG, MOG, and SEG citrus juice, respectively. These potent odorants mainly included 13–14 alcohols, 9–10 esters, 5 terpenes, 4 ketones, and 2 aldehydes. Fifteen odorants, including ethyl 2-methylbutyrate (OAV = 378,624.45), ethyl butyrate (OAV = 44,085.94), ethyl hexanoate (OAV = 37,730.80), hexanal (OAV = 4329.82), (+)-carvone (OAV = 2751.29), linalool (OAV = 1939.70), nonanal (OAV = 1810.95), hexyl acetate (OAV = 1076.69), a-terpineol (OAV = 818.27), β-myrcene (OAV = 740.31), a-ionone (OAV = 548.88), 3-methylbutanol (OAV = 414.96), a-pinene (OAV = 403.61), ethyl 3-hydroxyhexanoate (OAV = 344.85), and d-limonene (OAV = 335.99), were the important odorants that contributed to the characteristic aroma of HG citrus juice.

**Table 2:** OAVs of aroma compounds in both healthy and citrus juice with different CBS infection levels.

Compound	OAVs			
	HG	MIG	MOG	SEG
**Terpenes**				
α-pinene	404	447	36	701
β-myrcene	740	892	970	1408
p-1,3,8-menthatriene	131	100	148	235
d-limonene	336	371	376	488
β-caryophyllene	7	5	9	13
**Alcohols**				
2-methyl-3-buten-2-ol	3	<1	<1	<1
1-penten-3-ol	2	2	1	1
3-methylbutanol	415	445	412	366
2-methylbutanol	81	65	70	49
(Z)-3-hexenol	31	484	28	339
(E)-2-hexenol	18	29	6	40
hexanol	77	81	20	81
1-heptanol	255	195	181	196
1-octen-3-ol	107	399	422	707
1-octanol	10	55	56	94
linalool	1940	2908	5907	7763
terpinen-4-ol	<1	1	2	2
α-terpineol	818	1034	2060	2225
cis-carveol	<1	<1	1	1
citronellol	210	282	579	651
**Aldehydes**				
hexanal	4330	831	385	2871
nonanal	1811	1803	1957	2765
**Esters**				
ethyl acetate	2	2	2	2
methyl butanoate	28	21	33	28
ethyl isobutyrate	0	1227	663	998
ethyl butyrate	44,086	44,349	45,339	53,046
ethyl 2-methylbutyrate	378,624	478,594	670,178	655,629
methyl hexanoate	31	31	34	41
ethyl hexanoate	37,731	37,234	24,936	43,593
hexyl acetate	1077	447	483	1157
ethyl 3-hydroxyhexanoate	345	327	726	827
ethyl octanoate	185	131	58	68
**Ketones**				
6-methyl-5-hepten-2-one	22	25	37	25
(+)-carvone	2751	690	856	2246
α-ionone	549	461	458	449
geranylacetone	31	14	11	14

Note: The determined amounts of any compound are not the actual concentration due to the way SPME works. This is only reproducible when exact the same experimental procedure is followed.

When citrus fruits were infected with CBS, the OAVs of some odorants varied in the citrus juice. The OAVs of twelve odorants, namely, 6 alcohols (1-octen-3-ol, 1-octanol, linalool, terpinene-4-ol, α-terpineol and citronellol), 3 esters (ethyl isobutyrate, ethyl butyrate and ethyl 2-methylbutyrate), 2 terpenes (β-myrcene and d-limonene) and 1 ketone (6-methyl-5-hepten-2-one), were higher in the symptomatic groups than in the CBS-noninfected group. With the aggravation of infection, the OAVs of 9 odorants (β-myrcene, d-limonene, 1-octen-3-ol, 1-octanol, linalool, terpinen-4-ol, α-terpineol, citronellol, and ethyl butyrate) in citrus juice showed an increasing trend. β-Myrcene, with a high OAV (740.31–1407.98), was the predominant terpene odorant and contributed a pungent green and sour odor to citrus juice (Tu et al., 2002). Perez-Cacho showed that myrcene odor had a negative contribution to orange juice (Ruiz Perez-Cacho & Rouseff, 2008). The citrusy odor contribution of d-limonene to citrus juice improved when the citrus fruits were infected with CBS. Linalool and a-terpineol were the main alcohol odorants, accounting for approximately 48.64% ∼ 62.02% and 17.29% ∼ 21.14% of the total alcohol OAVs in citrus juice, respectively. Among the two odorants, linalool contributed to the floral odor of citrus juice; thus, our previous study showed that a-terpineol (floral, mint) induced an off-flavor in citrus juice (Cheng et al., 2022). Moreover, the highest OAVs were those of the esters with fruity smells, particularly ethyl 2-methylbutyrate (378,624.45–670,178.41), ethyl butyrate (44,085.94–53,045.53), and ethyl hexanoate (24,936.33–43,592.58) in citrus juice. High OAVs indicated that these odorants had a major contribution to the fruity characteristics of citrus juice, and changes in these esters may be related to the β-oxidation of precursor fatty acids (Idstein & Schreier, 1985). Moreover, ten odorants had a decreasing trend; that is, the OAVs of 10 odorants, namely, 4 alcohols (2-methyl-3-buten-2-ol, 1-penten-3-ol, 1-heptanol and 2-methylbutanol), 3 ketones ((+)-carvone, a-ionone, and geranylacetone), 2 esters (ethyl acetate and ethyl octanoate) and 1 aldehyde (hexanal), were lower in the symptomatic groups than in the CBS noninfected groups. Of the above odorants, only 3 (2-methyl-3-buten-2-ol, 1-penten-3-ol, and 1-heptanol) showed a decreasing trend at different infection severity levels in citrus juice. 2-Methyl-3-buten-2-ol, 1-penten-3-ol, 2-methylbutanol, hexanal and geranylacetone showed herbal/ green odors (https://www.vcf online.nl/VcfCompoundSearch.cfm). CBS infection decreased the herbal/ green sensory profile of citrus juice. Esters generally exhibit a fruity odor, and the increase in the OAVs of 3 esters (ethyl isobutyrate, ethyl butyrate, and ethyl 2-methylbutyrate) was higher than the decrease of the OAVs of 2 esters (ethyl acetate and ethyl octanoate). The fruity odor of the CBS-infected citrus juice sample was enhanced. As determined by comparing HG and SEG, the OAV of a-ionone decreased by 18.11%, while the OAV of linalool increased by 300.20%. In other words, CBS infection enhanced the floral profile in citrus juice.

Citrus essential oil in the CBS noninfected group contained 19 odorants, namely, 9 terpenes, 5 aldehydes, 2 alcohols, 2 esters, and 1 other (Table 3). In addition to camphene, other odorants were also detected in the essential oils of the symptomatic groups. Nine odorants, a-pinene (OAV = 65,585), sabinene (OAV = 6615), β-myrcene (OAV = 53,654), d-limonene (OAV = 14,610), linalool (OAV = 7663), nonanal (OAV = 6612), decanal (OAV = 2020), ethyl hexanoate (OAV = 108,223), and octyl acetate (OAV = 2282), were the main characteristic odors in the citrus essential oil of HG. When citrus fruits were infected with CBS, the OAVs of the odorants in the essential oil were higher than those in the HG group, such as sabinene (green), β-pinene (fresh, pine-like), β-myrcene (pungent green, sour), δ-3-carene (citrusy), d-limonene (pungent green, lemon-like), γ-terpinene (waxy), terpinolene (fresh, green), linalool (floral), citronellal (fresh, kabosu-like), octyl acetate (green, kabosu-like) and (*E*)-limonene oxide (floral, soapy) (Jia et al., 2022; Tu et al., 2002). The OAVs of camphene (medicine) (Cheng, Li, Wu, Huang, & Wang, 2021) had a decreasing trend when citrus fruits were infected with CBS. CBS improved the sensory profile of citrus essential oil, especially the characteristic odors of citrusy, green, and floral.

**Table 3:** OAVs of aroma compounds in both healthy and citrus essential oils with different CBS infection levels.

Compound	OAVs			
	HG	MIG	MOG	SEG
**Terpenes**				
α-pinene	65,585	72,769	115,096	63,876
camphene	113–130	71–82	0	0
sabinene	6615	7967	13,874	7056
β-pinene	116	133	178	124
β-myrcene	53,654	60,706	104,839	55,136
δ-3-carene	30	43	58	40
d-limonene	14,610	16,044	18,522	15,305
γ-terpinene	4	5	9	5
terpinolene	28	34	42	33
**Alcohols**				
1-octanol	24	19	34	8
linalool	7663	10,328	26,152	8883
**Aldehydes**				
nonanal	6612	9778	23,560	6452
citronellal	240	380	1010	312
decanal	2020	3164	2339	1665
neral	73	115	349	51
citral	65	98	369	43
**Esters**				
ethyl hexanoate	108,223	115,168	249,086	86,815
octyl acetate	2282	3250	3021	6081
**others**				
(E)-limonene oxide	13	19	65	42

Note: The determined amounts of any compound are not the actual concentration due to the way SPME works. This is only reproducible when exact the same experimental procedure is followed.

### 3.4 Correlation of aroma compounds with healthy or different CBS stages

**Fig. 3:**
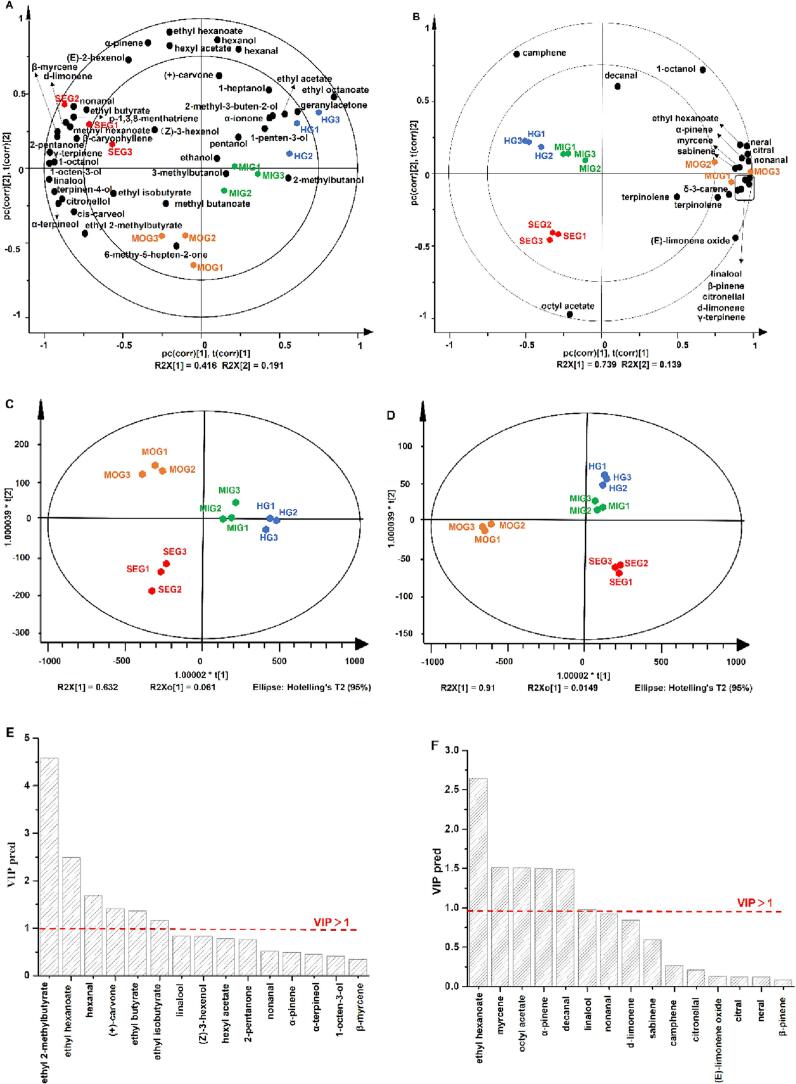
PCA and OPLS-DA results of OAV in both healthy and CBS-infected orange juice or essential oil, PCA biplots of orange juice (A) and essential oil (B), OPLS-DA score plot of orange juice (C) and essential oil (D), OPLS-DA VIP plot of orange juice (E) and essential oil (F).

Multivariate statistical analysis was applied to explore the relationships between odorants and different infected/noninfected citrus juice/essential oils and screen the markers associated with the infection levels of the citrus fruits. PCA and OPLS-DA were performed using four different CBS infected and noninfected citrus samples (one healthy group and three groups with different infection levels of citrus juice or essential oil) and the OAVs of each odorant. The juice and essential oil of four samples had the same distribution in the Fig. 3A and Fig. 3B. It also showed that there is a certain similarity in the changes of volatiles between citrus juice and essential oils. As shown in Fig.3A, the clusters of different citrus juice samples (HG, MIG, MOG, SEG) were separated in the biplot representation. Moreover, the distribution of variables indicates their contributions to juice samples. The contributions of 1-octanol, linalool, 1-octen-3-ol, terpinene-4-ol, a-terpineol, cis-carveol, citronellol, β-myrcene, p-1,3,8-menthatriene, d-limonene, r-terpinene, ethyl octanoate, methyl hexanoate, nonanal, and 2-pentanone to PC-1 were between 0.8 and 1.0, while those of hexanal, hexanol, a-pinene, ethyl hexanoate and hexyl acetate to PC-2 were between 0.8 and 1.0. Among these compounds, ethyl octanoate contributed to the X+ direction, and other odorants contributed to the X- direction. In particular, HG juice samples showed high correlations mainly with ethyl acetate, geranylacetone, ethyl acetate, 2-methyl-3-buten-2-ol, a-ionone, and 1-penten-3-ol. The MOG samples showed high correlations mainly with 6-methyl-5-hepten-2-one. The MIG samples were clustered on the positive axis of PC1 and highly associated with 3-methylbutanol and 2-methylbutanol. The SEG samples had high correlations with nonanal, ethyl butyrate, methyl hexanoate and terpene odorants. In Fig.3B, PC1 and PC2 accounted for 73.9% and 13.9% of the variance, respectively. The HG and MIG citrus essential oil samples were clustered closely on the negative axis of PC1 and the positive axis of PC2. The (HG and MIG) samples were completely separated from the MOG and SEG samples in space. The contribution of a-pinene, sabinene, β-pinene, myrcene, ethyl hexanoate, δ-3-carene, d-limonene, γ-terpinene, linalool, nonanal, (*E*)-limonene oxide, citronellal, neral, and citral to PC-1 reached 0.8 and 1.0, while that of octyl acetate to PC-2 reached 0.9 and 1.0. The MOG samples were clustered on the positive axis of PC1 and highly associated with most of the odorants in the citrus essential oil.

The OPLA-DA scores were graphed to reveal that the healthy citrus sample and different CBS infection levels had distribution characteristics. As shown in Fig.3C, the HG, MIG, MOG, and SEG citrus juice samples were divided into four groups to obtain OPLS-DA score plots. The HG and MIG samples were well separated from the other two groups (MOG and SEG) on the first principal component axis. The MOG and SEG samples were completely separated from the other two groups (MIG and HG) on the second principal component axis. However, there were differences in distribution between the citrus essential oil and citrus juice. In Fig.3D, the four citrus essential oil samples were divided into three camps along the second principal component. The HG citrus essential oil samples were not significantly separated from the MIG samples, indicating that there were no significant differences between the HG sample and the mild CBS infection samples (MIG). Furthermore, the principle of variable importance in projection (VIP) values >1 was used to further identify the most prominent variables and screen the markers (Mais et al., 2018). Six odorants were screened as discriminatory markers of different CBS infection levels in citrus juice, namely, ethyl 2-methylbutyrate, ethyl hexanoate, hexanal, (+)-carvone, ethyl butyrate and ethyl isobutyrate (Fig.3E). Five markers were screened in citrus essential oil samples with different CBS infection, namely, ethyl hexanoate, myrcene, octyl acetate, a-pinene and decanal (Fig.3F). Moreover, 200 permutation tests were conducted to judge whether the OPLS-DA model was overfitted. The intercepts R2 and Q2 were 0.451 and − 1.16, 0.307 and − 1.02, respectively. The results indicated that the OPLS-DA models are reliable and free of overfitting (Xue et al., 2022).

Although we have conducted a comprehensive study on flavor metabolites in citrus diseased fruits infected with CBS, the changes of nutritional components of these disease fruits, such as polyphenols, flavonoids, amino acids, etc., have not been explored. Therefore, a comprehensive study on the flavor and nutrition of citrus diseased fruits provides a scientific basis for evaluating the suitability of CBS citrus fruit processing. Moreover, in the process of utilizing CBS citrus fruits, the classification of citrus fruit infection degree is also very important. Currently, this classification is mainly based on experience, and more scientific methods should be explored to quantify the infection status and processing suitability of CBS fruits in the future.

## 4 Conclusion

To clarify the response of host citrus to CBS from the perspective of flavor, the concentration of volatiles in citrus juice and essential oils and their contribution to flavor were studied by using GC–MS combined with multivariate statistical methods. Fifty volatiles were positively identified in the infected or noninfected orange juice. There were 21, 21, 20, and 20 volatiles detected in the HG, MIG, MOG, and SEG essential oils, respectively. CBS disease resulted in a decrease in aldehyde and ketone contents and an increase in terpene and alcohol contents in citrus juice. The total volatile content in citrus juice and essential oil increased after infection with CBS disease, especially in the SEG juice and MOG essential oil, which reached the highest levels. Furthermore, CBS increased the OAVs of 9 odorants (β-myrcene, d-limonene, 1-octen-3-ol, 1-octanol, linalool, terpinen-4-ol, α-terpineol, citronellol and ethyl butyrate) and decreased those of 3 odorants (2-methyl-3-buten-2-ol, 1-penten-3-ol and 1-heptanol) at different CBS infection levels, leading to the enhance of floral, fruity and off-flavor aromas, and weak of green aroma. In citrus essential oil, CBS disease enhanced the OAVs of the eleven odorants, including sabinene, β-pinene, β-myrcene, δ-3-carene, d-limonene, γ-terpinene, terpinolene, linalool, citronellal, octyl acetate, and (*E*)-limonene oxide, leading to an increase in citrusy, floral and green aromas. Multivariate statistical analysis results showed that six odorants (ethyl 2-methylbutyrate, ethyl hexanoate, hexanal, (+)-carvone, ethyl butyrate, and ethyl isobutyrate) and five odorants (ethyl hexanoate, myrcene, octyl acetate, a-pinene and decanal) were screened as potential markers to characterize the change in aroma profile and the difference in infection levels in citrus juice and essential oil, respectively. These results indicated that the volatile concentration and aroma profiles of citrus juice and essential oil could reflect CBS infection and the different levels of CBS severity, providing a theoretical basis for the selection of raw materials for the processing of CBS infected citrus.

## CRediT authorship contribution statement

**Leng Han:** Writing – review & editing, Writing – original draft, Methodology, Formal analysis, Data curation, Conceptualization. **Guijie Li:** Writing – review & editing, Writing – original draft, Investigation, Formal analysis, Data curation, Conceptualization. **Xuting Wang:** Writing – review & editing, Formal analysis, Data curation. **Bo Yu:** Writing – review & editing, Data curation, Conceptualization. **Tenghui Zhang:** Writing – review & editing, Supervision, Conceptualization. **Yujiao Cheng:** Writing – review & editing, Visualization, Validation, Methodology, Data curation.

## Declaration of competing interest

There are no conflicts of interest to declare.

## Data Availability

Data will be made available on request.

## Appendix A Supplementary data

Supplementary material Image 1

## References

[bb0005] Aggarwal K.K., Khanuja S.P.S., Ahmad A., Santha Kumar T.R., Gupta V.K., Kumar S. (2002). Antimicrobial activity profiles of the two enantiomers of limonene and carvone isolated from the oils ofMentha spicata andAnethum sowa. Flavour and Fragrance Journal.

[bb0010] Benson A.H. (1895). Black spot of the orange. Agricultural Gazette of New South Wales.

[bb0015] Bora H., Kamle M., Mahato D.K., Tiwari P., Kumar P. (2020). Citrus essential oils (CEOs) and their applications in food: An overview. Plants (Basel).

[bb0020] Brentu F.C., Oduro K.A., Offei S.K., Odamtten G.T., Vicent A., Peres N.A., Timmer L.W. (2012). Crop loss, aetiology, and epidemiology of citrus black spot in Ghana. European Journal of Plant Pathology.

[bb0025] Buettner A., Schieberle P. (2001). Evaluation of aroma differences between hand-squeezed juices from valencia late and navel oranges by quantitation of key odorants and flavor reconstitution experiments. Journal of Agricultural and Food Chemistry.

[bb0030] Cheng Y.J., Li G.J., Wu H.J., Huang L.H., Wang H. (2021). Identification of light-induced key off-flavors in Ponkan mandarin juice using MDGC-MS/O and GC-MS/PFPD. Journal of Agricultural and Food Chemistry.

[bb0035] Cheng Y.J., Li G.J., Wu H.J., Liang G.L., Wang H. (2022). Flavor deterioration of mandarin juice during storage by MDGC-MS/O and GC-MS/PFPD. LWT- Food Science and Technology.

[bb0040] Chisholm M.G., Jell J.A., Cass D.M. (2003). Characterization of the major odorants found in the peel oil of Citrus reticulata Blanco cv. Clementine using gas chromatography-olfactometry. Flavour and Fragrance Journal.

[bb0045] (2024). Citrus: World markets and trade.

[bb0050] da Cruz M.A., Plotto A., Ferrarezi R.S., Leite Junior R.P., Bai J. (2023). Effect of Huanglongbing on the volatile organic compound profile of fruit juice and peel oil in “Ray Ruby” grapefruit. Foods.

[bb0055] Dagulo L., Danyluk M.D., Spann T.M., Valim M.F., Goodrich-Schneider R., Sims C., Rouseff R. (2010). Chemical characterization of orange juice from trees infected with citrus greening (Huanglongbing). Journal of Food Science.

[bb0060] Dala-Paula B.M., Plotto A., Bai J., Manthey J.A., Baldwin E.A., Ferrarezi R.S., Gloria M.B.A. (2018). Effect of Huanglongbing or greening disease on orange juice quality, a review. Frontiers in Plant Science.

[bb0065] EPPO (2020). https://gd.eppo.int/taxon/GUIGCI.

[bb0070] Futch H.S. (2011).

[bb0075] Han Y., Sun Z., Chen W. (2019). Antimicrobial susceptibility and antibacterial mechanism of limonene against listeria monocytogenes. Molecules.

[bb0080] Herman A., Tambor K., Herman A. (2016). Linalool affects the antimicrobial efficacy of essential oils. Current Microbiology.

[bb0085] Idstein H., Schreier P. (1985). Volatile constituents from guava (psidium-guajava, l) fruit. Journal of Agricultural and Food Chemistry.

[bb0090] Jia X., Ren J., Fan G., Reineccius G.A., Li X., Zhang N., Pan S. (2022). Citrus juice off-flavor during different processing and storage: Review of odorants, formation pathways, and analytical techniques. Critical Reviews in Food Science and Nutrition.

[bb0095] Kiely T.B. (1948). Preliminary studies on Guignardia citricarpa, n.sp.: The ascigerous stage of Phoma citricarpa McAlp. and its relation to black spot of Citrus. Proceedings of the Linnean Society of New South Wales.

[bb0100] Li G.J., Zhang Q.L., He Y.J., Tan A.Q., Zhang T.H., Guo L., Sun Z. (2020). Comparative analysis of volatile and principal aroma components of cold-pressed oil from three varieties of late-maturing sweet orange. Food and Frementation Industries.

[bb0105] Li Y.N., Zhang S.B., Lv Y.Y., Zhai H.C., Cai J.P., Hu Y.S. (2022). Linalool, the main volatile constituent from Zanthoxylum schinifolium pericarp, prevents growth of aspergillus flavus in post-harvest grains. Food Control.

[bb0110] Liu Y., He C., Song H. (2018). Comparison of SPME versus SAFE processes for the analysis of flavor compounds in watermelon juice. Food Analytical Methods.

[bb0115] Mais E., Alolga R.N., Wang S.L., Linus L.O., Yin X.J., Qi L.W. (2018). A comparative UPLC-Q/TOF-MS-based metabolomics approach for distinguishing Zingiber officinale roscoe of two geographical origins. Food Chemistry.

[bb0120] Malik A.U., Hasan M.U., Khalid S., Mazhar M.S., Shafique Khalid M., Khan M.N., Anwar R. (2021). Biotic and abiotic factors causing rind blemishes in citrus and management strategies to improve the cosmetic quality of fruits. International Journal of Agriculture and Biology.

[bb0125] Miles A.K., Smith M.W., Tran N.T., Shuey T.A., Dewdney M.M., Drenth A. (2019). Identification of resistance to citrus black spot using a Novel in-field inoculation assay. HortScience.

[bb0130] Moyo P., Fourie P.H., Masikane S.L., de Oliveira Fialho R., Mamba L.C., du Plooy W., Hattingh V. (2020). The effects of postharvest treatments and sunlight exposure on the reproductive capability and viability of phyllosticta citricarpa in citrus black spot fruit lesions. Plants (Basel).

[bb0135] Njoroge S.M., Ukeda H., Sawamura M. (1996). Changes in the volatile composition of yuzu (Citrus junos Tanaka) cold-pressed oil during storage. Journal of Agricultural and Food Chemistry.

[bb0145] Perez-Lopez A.J., Saura D., Lorente J., Carbonell-Barrachina A.A. (2006). Limonene, linalool, alpha-terpineol, and terpinen-4-ol as quality control parameters in mandarin juice processing. European Food Research and Technology.

[bb0150] Plotto A., Margaria C.A., Goodner K.L., Baldwin E.A. (2008). Odour and flavour thresholds for key aroma components in an orange juice matrix: Esters and miscellaneous compounds. Flavour and Fragrance Journal.

[bb0155] Rega B., Fournier N., Guichard E. (2003). Solid phase microextraction (SPME) of orange juice flavor: Odor representativeness by direct gas chromatography olfactometry (D-GC-O). Journal of Agricultural and Food Chemistry.

[bb0160] Ruiz Perez-Cacho P., Rouseff R.L. (2008). Fresh squeezed orange juice odor: A review. Critical Reviews in Food Science and Nutrition.

[bb0165] Schreuder W., du Plooy W., Erasmus A., Savage C., Basson E., Lennox C., Fourie P.H. (2018). Postharvest fungicide treatments and cold storage control citrus black spot infections. Crop Protection.

[bb0170] Shui M., Feng T., Tong Y., Zhuang H., Lo C., Sun H., Chen L., Song S. (2019). Characterization of key aroma compounds and construction of flavor base module of chinese sweet oranges. Molecules.

[bb0175] Silva Junior G.J., Feichtenberger E., Spósito M.B., Bassanezi R.B. (2016).

[bb0180] Singh B., Singh J.P., Kaur A., Yadav M.P. (2021). Insights into the chemical composition and bioactivities of citrus peel essential oils. Food Research International.

[bb0185] Tu N.T.M., Onishi Y., Choi H.S., Kondo Y., Bassore S.M., Ukeda H., Sawamura M. (2002). Characteristic odor components of Citrus sphaerocarpa Tanaka (Kabosu) cold-pressed peel oil. Journal of Agricultural and Food Chemistry.

[bb0190] UNECE (2010).

[bb0195] Wei C., Liu H., Cao X., Zhang M., Li X., Chen K., Zhang B. (2021). Synthesis of flavour-related linalool is regulated by PpbHLH1 and associated with changes in DNA methylation during peach fruit ripening. Plant Biotechnology Journal.

[bb0200] Wibowo S., Grauwet T., Gedefa G.B., Hendrickx M., Van Loey A. (2015). Quality changes of pasteurised mango juice during storage. Part II: Kinetic modelling of the shelf-life markers. Food Research International.

[bb0205] Xu B.M., Baker G.L., Sarnoski P.J., Goodrich-Schneider R.M. (2017). A comparison of the volatile components of cold pressed Hamlin and Valencia (Citrus sinensis (L.) Osbeck) orange oils affected by Huanglongbing. Journal of Food Quality.

[bb0210] Xu B.M., Sims C.A., Etxeberria E., Schneider R.M.G. (2017). Physicochemical and sensory properties of cold pressed oils from florida Hamlin and Valencia oranges affected by Huanglongbing. Journal of Food Science.

[bb0215] Xue W.F., Zhang H., Wang M., Liu Y., Liu M., Shen B. (2022). Metabolomics-based non-targeted screening analysis of 34 PPCPs in bovine and piscine muscles. Analytical Methods.

[bb0220] Yu H., Lin Z.X., Xiang W.L., Huang M., Tang J., Lu Y., Liu L. (2022). Antifungal activity and mechanism of D-limonene against foodborne opportunistic pathogen Candida tropicalis. LWT- Food Science and Technology.

