# Supplementary material for: Characterization of volatile compounds from healthy and citrus black spot-infected Valencia orange juice and essential oil by using gas chromatography–mass spectrometry
Source: Food Chem X. 2024 Apr 10;22:101374. doi: 10.1016/j.fochx.2024.101374 (PMC11052904; doi:10.1016/j.fochx.2024.101374)
Supplement: Supplementary file 1 — Supplementary material [file mmc1.pdf]

SUPPLEMENTARY

Characterization of volatile compounds from healthy and citrus black spot-infected orange juice and essential oil by using gas chromatography–mass spectrometry

Leng Han[1,2#], Guijie Li[1,2#], Xuting Wang[1], Bo Yu[3], Tenghui Zhang[4], Yujiao Cheng*[1,2]

[1] Citrus Research Institute, Southwest University, Chongqing 400712, China.

[2] National Citrus Engineering Research Center, Chinese Academy of Agricultural Sciences, Chongqing 400712, China

[3] Sichuan Dan Orange Modern Fruit Industry Co., Ltd., Sichuan 620200, China

[4] Chongqing Centre Testing International Group Co., Ltd., Chongqing 400712, China

Emails: hanleng@cric.cn, liguijie@cric.cn,

# Leng Han and Guijie Li contribute equally.

*Corresponding author email= chengyujiao1@cric.cn

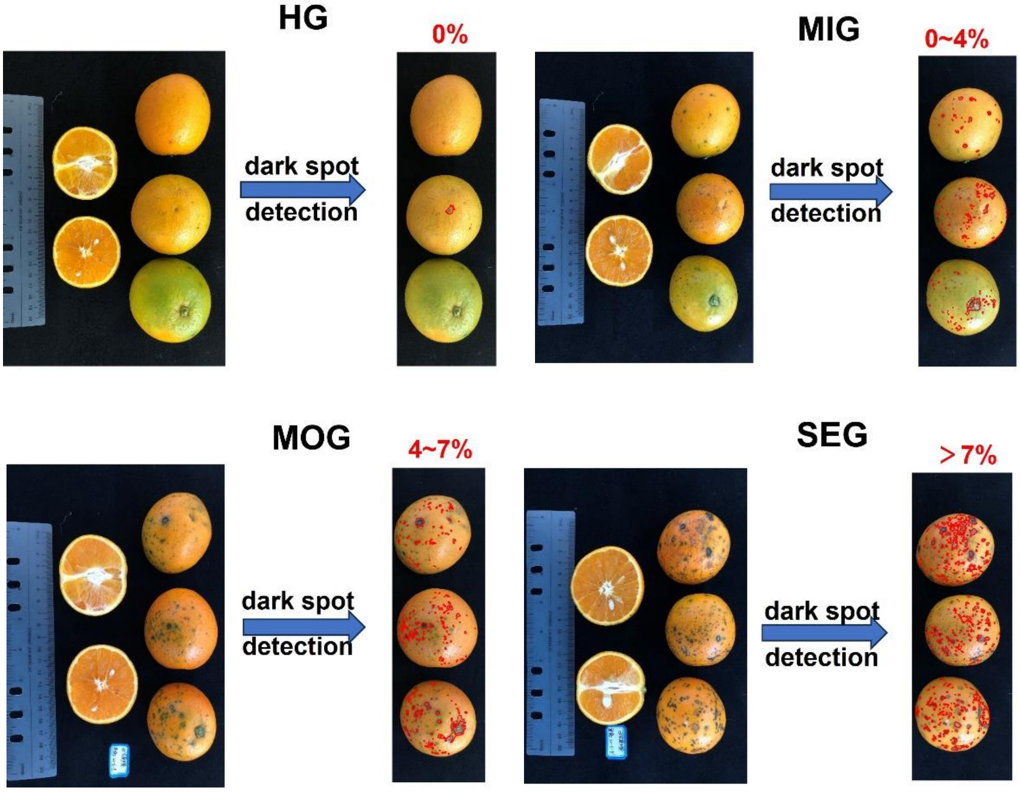

Fig. S1. Classification photographs of different black spot infection degree of citrus fruits according to the proportion of the area of the dark spot to the total area

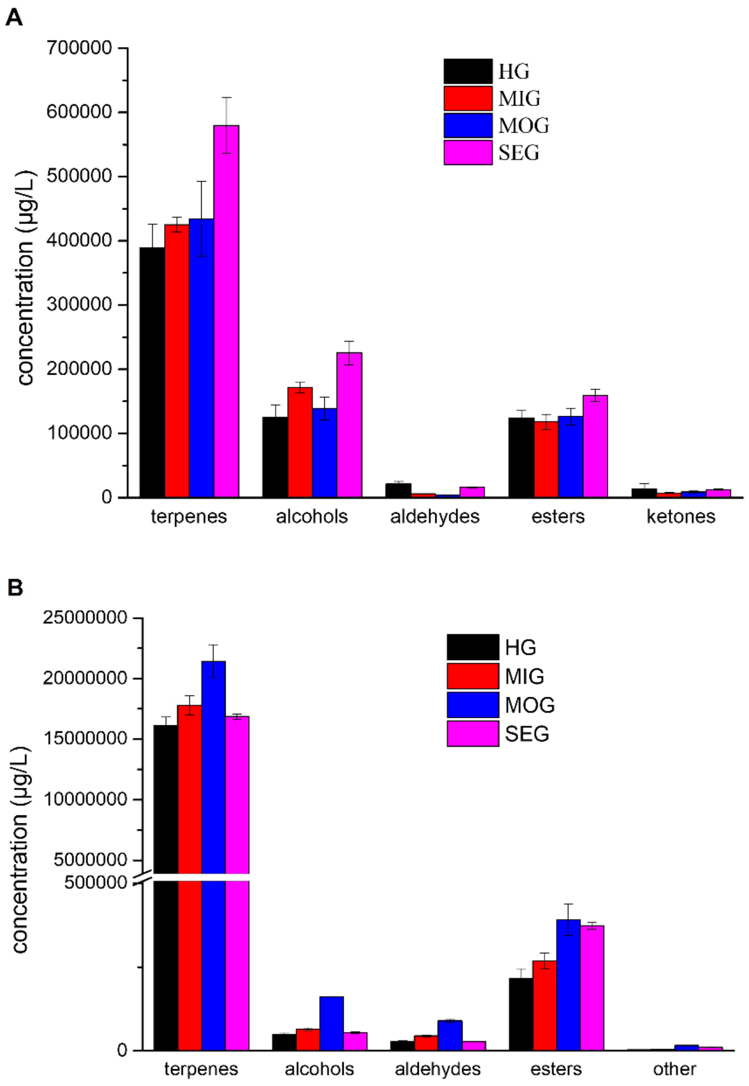

Fig. S2. Changes in VOCs in both healthy and black spot orange juice (A) and essential oil (B)

Table S1. OAVs of aroma compounds in both healthy and citrus juice with different CBS infection levels

Compound	Odor threshold[w] (μg/L)	Reference
**Terpenes**		
α-pinene	6	(Pino & Mesa, 2006)
β-myrcene	15	(Pino & Mesa, 2006)
p-1,3,8-menthatriene	15	(Masanetz & Grosch, 1998)
d-limonene	1000	(Tamura, Fukuda, & Padrayuttawat, 1996)
β-caryophyllene	64	(Pino & Mesa, 2006)
camphene	26-30[a]	(Stone, Pryor, & Steinmetz., 1972)
sabinene	37	(Pino, Torricella, & Orsi, 1986)
β-pinene	140	(Pino & Mesa, 2006)
δ-3-carene	770	(Pino & Mesa, 2006)
γ-terpinene	1000	(Tamura, Fukuda, & Padrayuttawat, 1996)
terpinolene	200	(Pino & Mesa, 2006)
**Alcohols**		
2-methyl-3-buten-2-ol	1140	(Boonbumrung et al., 2001)
1-penten-3-ol	358.1	(Giri, Osako, & Ohshima, 2010)
3-methylbutanol	4	(Giri, Osako, & Ohshima, 2010)
2-methylbutanol	15.9	(Giri, Osako, & Ohshima, 2010)
(Z)-3-hexenol	70	(Hansen, Buttery, Stern, Cantwell, & Ling, 1992)
(E)-2-hexenol	400	(Hansen, Buttery, Stern, Cantwell, & Ling, 1992)
hexanol	500	(Hansen, Buttery, Stern, Cantwell, & Ling, 1992)
1-heptanol	5.4	(Giri, Osako, & Ohshima, 2010)
1-octen-3-ol	1.5	(Giri, Osako, & Ohshima, 2010)
1-octanol	125.8	(Giri, Osako, & Ohshima, 2010)
linalool	6	(Pino & Mesa, 2006)
terpinen-4-ol	6400	(Tamura, Yang, & Sugisawa, 1993)
α-terpineol	4.6	(Pino, Torricella, & Orsi, 1986)
cis-carveol	4000	(Tamura, Yang, & Sugisawa, 1993)
citronellol	10.6	(Pino, Torricella, & Orsi, 1986)
**Aldehydes**		
hexanal	4.5	(Hansen, Buttery, Stern, Cantwell, & Ling, 1992)
nonanal	1.1	(Giri, Osako, & Ohshima, 2010)
citronellal	31	(Pino, Torricella, & Orsi, 1986)
decanal	4.9	(Pino, Torricella, & Orsi, 1986)
neral	30	(Buttery, Teranishi, Flath, & Ling, 1989)
citral	28	(Pino, Torricella, & Orsi, 1986)
**Esters**		
ethyl acetate	5000	(Hansen, Buttery, Stern, Cantwell, & Ling, 1992)
methyl butanoate	59	(Pino & Mesa, 2006)
ethyl isobutyrate	0.1	(Pino & Mesa, 2006)

ethyl butyrate	1.1	(Pino, Torricella, & Orsi, 1986)
ethyl 2-methylbutyrate	0.006	(Pino & Mesa, 2006)
methyl hexanoate	70	(Pino & Mesa, 2006)
ethyl hexanoate	1	(Hansen, Buttery, Stern, Cantwell, & Ling, 1992)
hexyl acetate	2.3[a]	(Stone, Pryor, & Steinmetz., 1972)
ethyl 3-hydroxyhexanoate	45[b]	(Moyano, Zea, Moreno, & Medina, 2010)
ethyl octanoate	19.3	(Giri, Osako, & Ohshima, 2010)
octyl acetate	47	(Pino & Mesa, 2006)
**Ketones**		
6-methyl-5-hepten-2-one	68	(Giri, Osako, & Ohshima, 2010)
(+)-carvone	2.7	(Ahmed, Dennison, Dougherty, & Shaw, 1978)
α-ionone	0.6	(Buttery & Ling, 1995)
geranylacetone	60	(Buttery, Teranishi, Flath, & Ling, 1989)
**others**		
(E)-limonene oxide	250	(Tamura, Yang, & Sugisawa, 1993)

a=odor threshold in air, w=odor threshold in water, b=odor threshold in 14% V/V ethanol/water

Reference：

Ahmed, E.M., Dennison, R.A., Dougherty, R.H., & Shaw, P.E. (1978). Flavor and odor thresholds in water of selected orange juice components. *Journal of Agricultural and Food Chemistry, 26*(1), 187-191. https://doi.org/10.1021/jf60215a074.

Boonbumrung, S., Tamura, H., Mookdasanit, J., Nakamoto, H., Ishihara, M., Yoshizawa, T., & Varanyanond, W. (2001). Characteristic Aroma Components of the Volatile Oil of Yellow Keaw Mango Fruits Determined by Limited Odor Unit Method. *Food Science & Technology International Tokyo, 7*(3), 200-206. https://doi.org/10.3136/fstr.7.200.

Buttery, R.G., & Ling, L.C. (1995). Volatile flavor components of corn tortillas and related products. *Journal of Agricultural and Food Chemistry, 43*(7), 5. https://doi.org/10.1021/jf00055a023.

Buttery, R.G., Teranishi, R., Flath, R.A., & Ling, L.C. (1989). Fresh tomato volatiles - composition and sensory studies. In Teranishi R., Buttery R. & Shahidi F. (Eds.), *Flavor Chemistry: Trends and Developments* (p. 9). Washington: American Chemical Society.

Giri, A., Osako, K., & Ohshima, T. (2010). Identification and characterisation of headspace volatiles of fish miso, a Japanese fish meat based fermented paste, with special emphasis on effect of fish species and meat washing. *Food Chemistry, 120*(2), 621-631. https://doi.org/10.1016/j.foodchem.2009.10.036.

Hansen, M., Buttery, R.G., Stern, D.J., Cantwell, M.I., & Ling, L.C. (1992). Broccoli storage under low-oxygen atmosphere - identification of higher boiling volatiles. *Journal of Agricultural and Food Chemistry, 40*(5), 850-852. https://doi.org/10.1021/jf00017a029.

Masanetz, C., & Grosch, W. (1998). Key odorants of parsley leaves (Petroselinum crispum Mill. Nym. ssp. crispum) by odour-activity values. *Flavour and Fragrance Journal, 13*(2), 115-124. https://doi.org/10.1002/(sici)1099-1026(199803/04)13:2.

Moyano, L., Zea, L., Moreno, J.A., & Medina, M. (2010). Evaluation of the active odorants in Amontillado sherry wines during the aging process. *Journal of Agricultural and Food Chemistry, 58*(11), 6900-6904. https://doi.org/10.1021/jf100410n.

Pino, J., Torricella, R., & Orsi, F. (1986). Correlation between sensory and gas-chromatographic measurements on grapefruit juice volatiles. *Nahrung-Food, 30*(8), 7. https://doi.org/10.1007/BF01087743.

Pino, J.A., & Mesa, J. (2006). Contribution of volatile compounds to mango (Mangifera indica L.) aroma. *Flavour and Fragrance Journal, 21*(2), 207-213. https://doi.org/10.1002/ffj.1703.

Stone H., Pryor G.T., & Steinmetz G. (1972). A comparison of olfactory adaptation among seven odorants and their relationship with several physiochemical properties. *Perception & Psychophysics, 12*, 501-504. https://doi.org/10.3758/bf03210944.

Tamura H, Fukuda Y, & Padrayuttawat A. (1996). Characterization of citrus aroma quality by odor threshold values. In Takeoka G. R., Teranishi R., Williams P. J. & Kobayashi A. (Eds.), *Biotechnology for Improved Foods and Flavors* (pp. 282-294). Washington: American Chemical Society.

Tamura, H., Yang, R.H., & Sugisawa, H. (1993). Aroma profiles of peel oils of acid citrus. *Acs Symposium Series, 525*, 121-136. https://doi.org/10.1021/bk-1993-0525.ch010.
